# Uterine intermural adenosarcoma arising from adenomyoma: A case report and review of literature

**DOI:** 10.1097/MD.0000000000043829

**Published:** 2025-08-29

**Authors:** Jingwen Qiu, Li Yuan

**Affiliations:** a Department of Gynecology and Obstetrics, Nanjing Lishui People’s Hospital, Nanjing, China.

**Keywords:** immunohistochemistry, uterine adenomyoma, uterine intermural adenosarcoma

## Abstract

**Rationale:**

Intramural adenosarcoma of the uterine myometrium is an uncommon gynecologic malignancy, often diagnosed postoperatively, and may arise from pre-existing benign lesions such as adenomyoma.

**Patient concerns:**

A 46-year-old woman presented with irregular vaginal bleeding during the postmenstrual period for 3 months. She had a prior history of adenomyoma resection and breast cancer treated with tamoxifen.

**Diagnoses:**

Pelvic ultrasonography suggested multiple uterine myomas. Histopathological and immunohistochemical analyses of the resected specimen confirmed low-grade malignant uterine adenosarcoma originating from adenomyoma.

**Interventions:**

The patient underwent a lower abdominal hysterectomy with bilateral adnexectomy.

**Outcomes:**

Postoperative recovery was uneventful. No adjuvant therapy was administered, and the patient remains under regular oncology follow-up without recurrence to date.

**Lessons:**

This case highlights the potential for malignant transformation of adenomyoma into intramural adenosarcoma and underscores the importance of thorough histopathological evaluation in patients with recurrent or atypical uterine masses.

## 1. Introduction

Intramural uterine adenosarcoma is a rare gynecologic malignancy arising from glandular tissue within the myometrium.^[1]^ Uterine adenosarcoma (UA) is a subtype of uterine sarcoma, accounting for 5% to 10% of all uterine sarcoma cases.^[2–4]^ The diagnosis typically relies on postoperative pathological examination.^[5]^ While macroscopic features are highly variable, microscopic analysis reveals biphasic differentiation, with both malignant stromal and benign epithelial components.^[6]^ Intramural UA is characterized by aggressive growth^[7]^ and significant heterogeneity,^[8]^ although its underlying etiology and pathogenesis remain poorly understood.

We present a case of uterine intramural adenosarcoma, with emphasis on its diagnosis, treatment, and recent clinical insights.

## 2. Case presentation

A 46-year-old woman, gravida IV para I, presented to the gynecology outpatient clinic with irregular vaginal bleeding during the postmenstrual period for the past 3 months. She reported regular menstrual cycles of 28 to 30 days, lasting 5 to 7 days.

Her medical history revealed the incidental discovery of a 2.0 cm uterine fibroid during a physical examination 11 years ago, for which she received no treatment. Five years ago, due to increased size and number of fibroids, she underwent an abdominal myomectomy with removal of 13 fibroids. Pathological examination confirmed adenomyoma. Postoperative gynecological follow-up was performed irregularly. Three years ago, she underwent surgery for breast cancer and has since been taking oral tamoxifen.

Gynecological examination showed mild displacement of columnar epithelium on the cervix. The uterus was irregularly enlarged, consistent with a size typical of a 4-month pregnancy, with medium firmness and palpable protrusions on both anterior and posterior walls.

**Figure 1.**
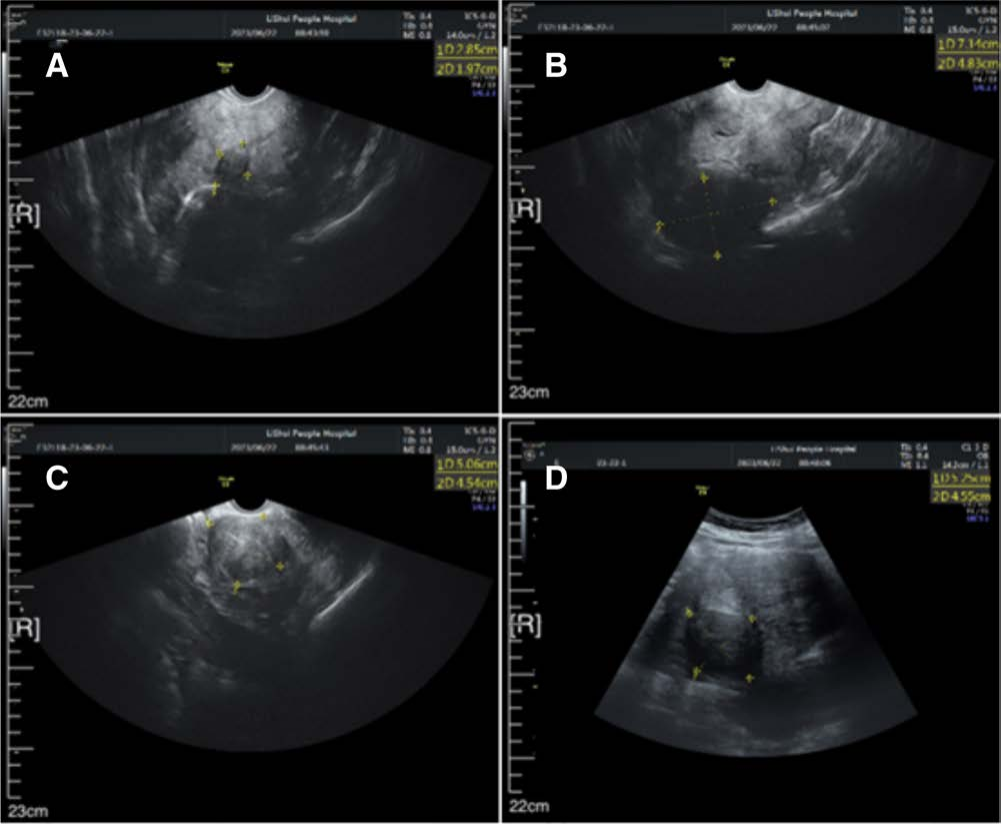
Transvaginal ultrasonography images. (A) Hypoechoic mass in the anterior wall of the uterus, about 29 × 20 mm in size; (B) Myomatoid mass fused with the posterior wall of uterus, 71 × 84 mm in size. (C, D) Another myomatoid mass located in the posterior wall, 51 × 46 mm in size.

Transvaginal ultrasonography (Fig. 1) revealed multiple heterogeneous echogenic masses within the myometrium, as well as several hypoechoic lesions in the uterine corpus, consistent with multiple uterine myomas.

Liquid-based thin-layer cytology of the cervix was unremarkable. Uterine curettage showed proliferative endometrium with endometrial polyp formation and intramural hemorrhage. Serum tumor markers were within normal limits, except for a mild elevation in cancer antigen 125 (CA125) at 48.6 U/mL (reference range: 0–35 U/mL).

**Figure 2.**
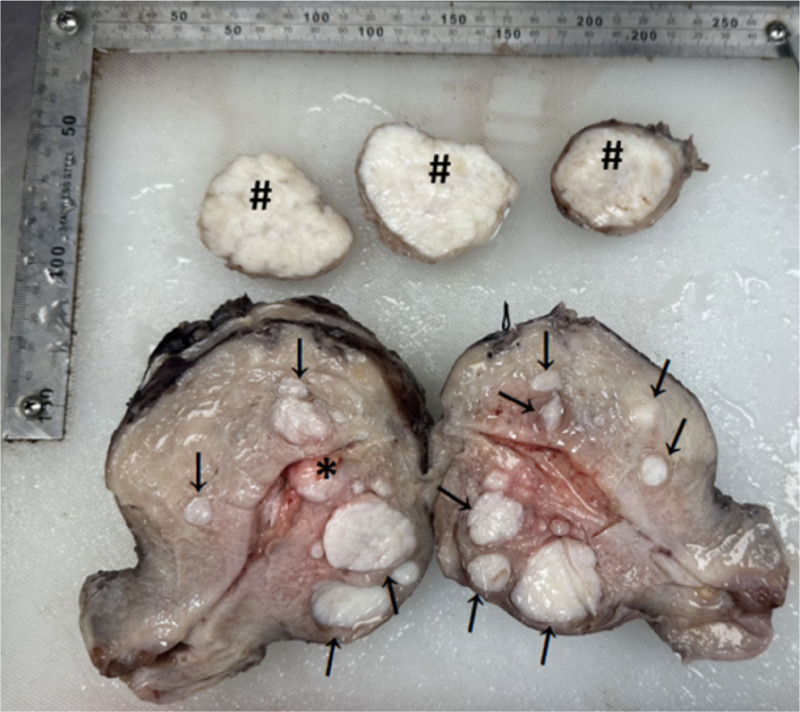
Macroscopic surgical specimen of uretes. ↓ indicates intermural fibroids, * indicates a submucosal fibroid, and # indicates subserous fibroids that have been removed.

**Figure 3.**
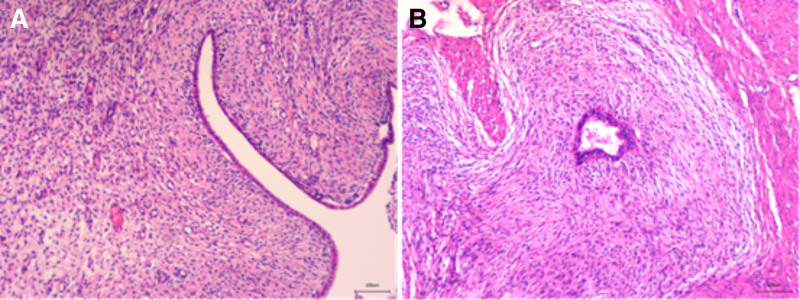
The hematoxylin–eosin staining of the specimen shows (A) papillary structure; and (B) sleeve-like structure.

**Figure 4.**
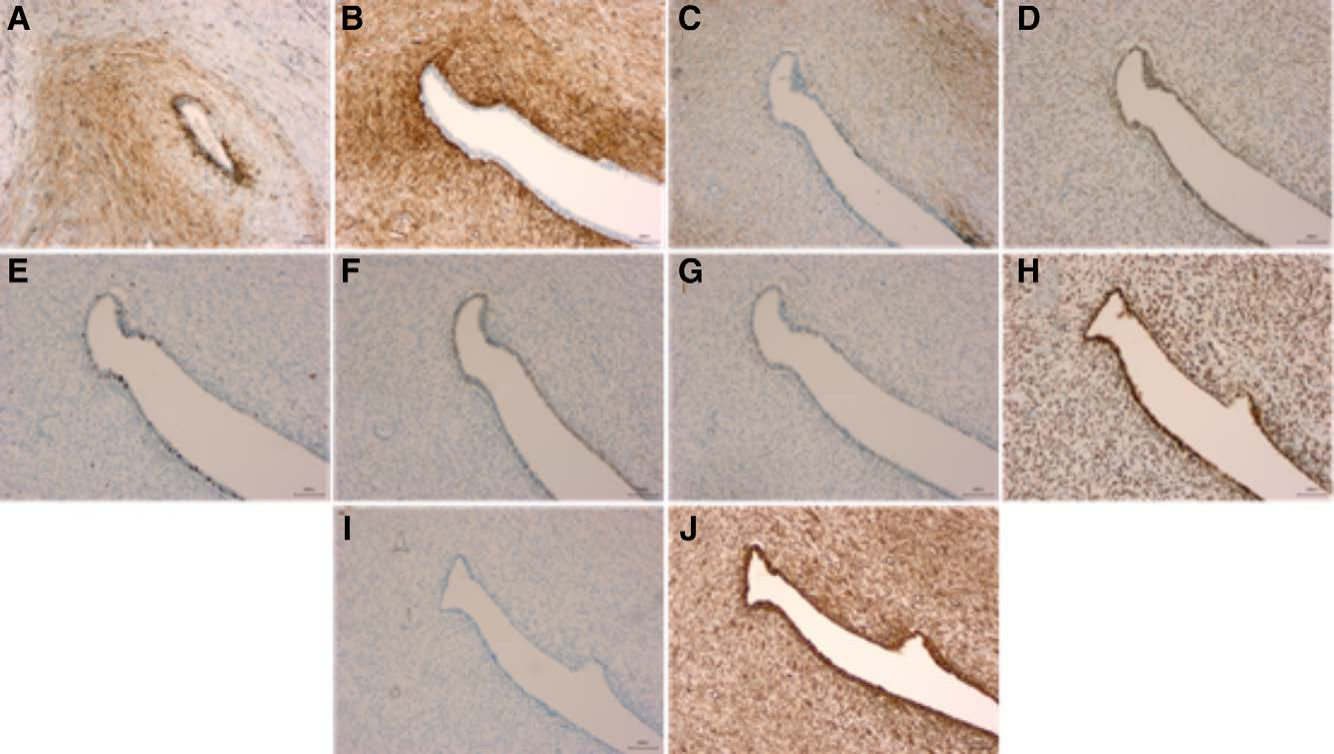
The immunohistochemistry of specimens for different antibody expression, (A) CD10; (B) CD34; (C) Desmin; (D) ER; (E) Ki67; (F) P16; (G) P53; (H) PR; (I) SMA; and (J) Vimentin.

The patient underwent a lower abdominal hysterectomy with bilateral adnexectomy under general anesthesia. Gross examination of the resected uterus (Fig. 2) revealed a uterine body measuring 10 × 8.5 × 9.5 cm, with a uterine cavity depth of 7.5 cm and endometrial thickness of 0.1 cm. A single endometrial polyp measuring 2 × 1.7 × 0.6 cm was noted. Over 20 fibroids were identified within the uterine wall, ranging from 0.3 to 4.5 cm in diameter. Three subserosal leiomyomas were also removed, ranging from 3.7 to 5.5 cm. All fibroids were solid, grayish-white, braided, and firm in texture. A grayish-yellow, leiomyoma-like lesion measuring approximately 3.5 cm with a firm consistency was observed in the uterine wall. Histopathological examination (Fig. 3) demonstrated mixed epithelial and mesenchymal tumors in the myometrium and endometrium, with classic papillary and sleeve-like structures on hematoxylin–eosin staining. Immunohistochemical analysis (Fig. 4) showed CD10 (mesenchymal cells +), CD34 (mesenchymal cells +), Desmin (−), ER (+), Ki67 (<5%), P16 (−), P53 (+, wild type), PR (+), SMA (−), and Vimentin (+). The final pathological diagnosis was low-grade malignant UA.

The patient was discharged postoperatively and has been under regular oncology follow-up without receiving adjuvant therapy.

## 3. Discussion

UA can occur in women aged 15 to 90 years, with most cases diagnosed in postmenopausal women.^[9]^ The median age of onset is 50 to 59 years, and recent studies indicate a trend toward younger patients.^[10]^ The etiology and pathogenesis of UA remain unclear. Previous studies have suggested that endometriosis may be a risk factor for extrauterine adenosarcoma, and that tamoxifen use may increase the risk of uterine carcinoma.^[11]^ Additional risk factors include prior radiation exposure, comorbid diabetes,^[12]^ tamoxifen-treated breast cancer,^[13,14]^ and recurrent cervical or endometrial polyps.^[15]^ The patient in this case had a history of uterine adenomyomas and was diagnosed with breast cancer 3 years ago. She has been taking oral tamoxifen since her breast surgery—factors considered high risk for the development of this tumor. Recent studies have also identified associations between uterine adenosarcoma and both estrogen and progesterone levels,^[16]^ as well as specific gene mutations,^[17]^ offering new directions for research on its pathogenesis.

UA commonly grows as an exophytic polyp in the uterine cavity and may also arise in the lower uterine segment, endocervical canal, or even extrauterine sites. Clinical symptoms include abnormal vaginal bleeding, lower abdominal pain, and tumor protrusion. Abnormal vaginal bleeding is the most frequent symptom, occurring in approximately 70% of patients.^[18]^

UA is often suspected based on gynecological examination, imaging, diagnostic curettage, and hysteroscopy, but definitive diagnosis requires pathological and immunohistochemical confirmation.

Ultrasound is the preferred imaging modality for evaluating myometrial lesions in women. Benign uterine leiomyomas typically appear as well-defined masses with a distinct capsule and peripheral vascular patterns. In contrast, adenomyosis lacks a capsule and exhibits more prominent internal vascularity.^[19]^ Features suggestive of malignancy include irregular myometrial thickening, hypoechoic areas, ill-defined tumor margins, heterogeneous echotexture, cystic components, and increased vascularity.^[20]^ A study of 2068 female patients developed a color-coded diagnostic algorithm that integrates clinical symptoms and ultrasound features of myometrial lesions. This approach provides guidance for personalized management of patients with suspected myometrial pathology.^[21]^

Magnetic resonance imaging (MRI) is advantageous for soft tissue imaging. Diffusion-weighted imaging (DWI) and contrast-enhanced MRI are frequently used for diagnosing and differentiating uterine sarcomas.^[22,23]^ The application of DWI and apparent diffusion coefficient values significantly enhances diagnostic accuracy.^[24]^ Antonio et al analyzed 8 studies comparing leiomyomas and sarcomas, reporting sensitivity, specificity, and area under the curve values of 0.90, 0.96, and 0.9759, respectively.^[25]^ Francesca et al confirmed that multiparametric MRI is useful for differentiating uterine mesenchymal tumors, with DWI being the most sensitive parameter and apparent diffusion coefficient values showing strong diagnostic relevance.^[24]^ When indicated, positron emission tomography/computed tomography should be considered to evaluate metastasis in high-risk patients.

Macroscopically, UA typically appears as a soft, lobulated, polypoid mass located within the uterine cavity.^[3]^ Although most tumors originate in the endometrium, some may arise from the endocervix or myometrium. Tumor sizes range widely from 1 cm to over 10 cm.^[26]^ Necrosis and hemorrhage may be observed in some cases.^[27]^ In the present case, the UA measured between 0.3 and 4.5 cm; however, no areas of hemorrhage or necrosis were identified.

Histologically, UA is characterized as a biphasic tumor, with benign epithelial glands and malignant sarcomatoid stromal cells. These stromal cells typically encircle the endometrial glands in a “cuff-like” arrangement.^[28]^ Approximately one-quarter of UAs contain heterologous mesenchymal elements, most commonly with rhabdomyosarcomatous differentiation. In this case, papillary and sleeve-like structures were observed microscopically, consistent with the malignant features of adenosarcoma and resembling the cuff-like pattern.

Early diagnosis enables timely medical intervention, such as hormone therapy, to alleviate symptoms and prevent disease progression. In patients with uterine leiomyomas, it also facilitates monitoring of tumor growth and allows early identification of malignant transformation risk. Moreover, early diagnosis helps avoid or delay surgical intervention, supports the medical management of severe symptoms, and preserves fertility when desired. Advances in noninvasive imaging techniques, including ultrasound and MRI, have improved the detection of adenomyosis in younger patients, thereby enabling earlier treatment.^[19]^

There are no specific serologic markers for uterine sarcoma, though mild to moderate elevations in cancer antigen 125 (CA125)^[29]^ and cancer antigen 19-9 (CA19-9)^[30]^ have been reported. Some studies suggest that serum lactate dehydrogenase (LDH)^[31]^ may aid in diagnosis. Li et al found that combining MRI with LDH testing improved sensitivity and specificity to 100%.^[32]^ Nishigaya et al further demonstrated that preoperative serum levels of LDH, d-dimer, and C-reactive protein can assist in differentiating uterine fibroids, particularly in cases of degenerative or atypical fibroids.^[33]^

Although adenomyosis is typically a benign condition, there is a rare risk of malignant transformation, even in young patients.^[19]^ Clinical signs such as rapid tumor growth or abnormal bleeding, along with ultrasound features like irregular margins, heterogeneous echotexture, and increased vascularity, should raise suspicion for malignancy.^[20]^ Regular follow-up with imaging is essential in young patients to ensure early detection of malignant changes, enabling timely intervention and reducing the risk of adverse outcomes.^[21]^

Management depends on the patient’s age, symptom severity, and reproductive goals. The main treatment options for UA include surgery, radiotherapy, and chemotherapy.^[34,35]^ Combination therapy with radiotherapy and chemotherapy may improve outcomes. Emerging treatments, including targeted therapy and immunotherapy, are gradually being introduced.^[36,37]^

First-line treatments typically include medical therapies such as continuous oral contraceptives and gonadotropin-releasing hormone agonists, which suppress hormonal stimulation and relieve symptoms.^[19]^ Despite these advances, surgical resection remains the standard treatment,^[38]^ typically consisting of total hysterectomy with bilateral adnexectomy. The role of pelvic lymph node dissection remains controversial.^[28]^ In patients requiring surgery, a multidisciplinary approach ensures optimal management and follow-up.^[21]^ For younger patients, fertility preservation must be carefully considered when selecting a surgical approach. Even after symptom relief, regular follow-up remains necessary to monitor for disease recurrence or transformation.^[19]^ Hormone therapy may be appropriate for inoperable or metastatic low-grade adenosarcoma that expresses estrogen and progesterone receptors. In contrast, doxorubicin-based chemotherapy is often recommended for high-grade tumors, although its efficacy is still under investigation.^[38]^

The prognosis of UA depends on tumor stage, histological features, and treatment strategy.^[39]^ Myometrial invasion and sarcomatous overgrowth are major prognostic factors. Sarcomatous overgrowth—defined as malignant stromal components comprising more than 25% of the tumor—occurs in approximately 10% of cases.^[4]^ The 5-year survival rate is 70% to 80% in patients without sarcomatous overgrowth but decreases to 50% to 60% in those with overgrowth.^[3]^ Additional research is necessary to identify prognostic biomarkers and to develop clinical models for improved patient management. In this case, a structured follow-up plan has been implemented and will continue.

## 4. Conclusion

This report describes a case of uterine intramural adenosarcoma arising from adenomyoma, and provides a review of its clinical features, diagnostic approach, treatment strategies, and prognosis.

## Author contributions

**Conceptualization:** Li Yuan.

**Data curation:** Jingwen Qiu.

**Formal analysis:** Jingwen Qiu.

**Investigation:** Jingwen Qiu.

**Methodology:** Jingwen Qiu.

**Project administration:** Li Yuan.

**Resources:** Li Yuan.

**Software:** Li Yuan.

**Supervision:** Li Yuan.

**Validation:** Li Yuan.

**Visualization:** Li Yuan.

**Writing – original draft:** Jingwen Qiu, Li Yuan.

**Writing – review & editing:** Jingwen Qiu, Li Yuan.
